# Phospholipase D1 and D2 Synergistically Regulate Thrombus Formation

**DOI:** 10.3390/ijms21186954

**Published:** 2020-09-22

**Authors:** Li-Ming Lien, Wan-Jung Lu, Ting-Yu Chen, Tzu-Yin Lee, Hsueh-Hsiao Wang, Hsien-Yu Peng, Ray-Jade Chen, Kuan-Hung Lin

**Affiliations:** 1 Department of Neurology, School of Medicine, College of Medicine, Taipei Medical University, Taipei 110, Taiwan; M002177@ms.skh.org.tw; 2 Department of Neurology, Shin Kong Wu Ho-Su Memorial Hospital, Taipei 111, Taiwan; 3 Department of Pharmacology, School of Medicine, College of Medicine, Taipei Medical University, Taipei 110, Taiwan; luwj@tmu.edu.tw (W.-J.L.); y0513260323@tmu.edu.tw (T.-Y.C.); 4 Department of Medical Research, Taipei Medical University Hospital, Taipei 110, Taiwan; 5 Graduate Institute of Metabolism and Obesity Sciences, College of Nutrition, Taipei Medical University, Taipei 110, Taiwan; 6 Department of Surgery, School of Medicine, College of Medicine, Taipei Medical University, Taipei 110, Taiwan; 7 Graduate Institute of Medical Sciences, College of Medicine, Taipei Medical University, Taipei 110, Taiwan; d119103001@tmu.edu.tw; 8 Department of Medicine, MacKay Medical College, New Taipei City 252, Taiwan; okul.wang@gmail.com (H.-H.W.); Hsien.Yu@gmail.com (H.-Y.P.); 9 Division of General Surgery, Department of Surgery, Taipei Medical University Hospital, 110 Taipei, Taiwan; 10 Institute of Biomedical Sciences, MacKay Medical College, New Taipei City 252, Taiwan

**Keywords:** phospholipase D, platelet, thrombosis, middle cerebral artery occlusion

## Abstract

Previously, we reported that phospholipase D1 (PLD1) and PLD2 inhibition by selective PLD1 and PLD2 inhibitors could prevent platelet aggregation in humans, but not in mice. Moreover, only the PLD1 inhibitor, but not PLD2 inhibitor, could effectively prevent thrombus formation in mice, indicating that PLD might play different roles in platelet function in humans and mice. Although PLD1 and PLD2 were reported to be implicated in thrombotic events, the role of PLD in mice remains not completely clear. Here, we investigated the role of PLD1 and PLD2 in acute pulmonary thrombosis and transient middle cerebral artery occlusion-induced brain injury in mice. The data revealed that inhibition of PLD1, but not of PLD2, could partially prevent pulmonary thrombosis-induced death. Moreover, concurrent PLD1 and PLD2 inhibition could considerably increase survival rate. Likewise, inhibition of PLD1, but not PLD2, partially improved ischemic stroke and concurrent inhibition of PLD1, and PLD2 exhibited a relatively better protection against ischemic stroke, as evidenced by the infarct size, brain edema, modified neurological severity score, rotarod test, and the open field test. In conclusion, PLD1 might play a more important role than PLD2, and both PLD1 and PLD2 could act synergistically or have partially redundant functions in regulating thrombosis-relevant events.

## 1. Introduction

Nowadays, a number of researchers or scientists are dedicated to discover a novel drug or new therapeutic strategy that not only prevents thrombosis-relevant events, such as stroke and heart attack, but also has minimal bleeding effect. Moreover, several potential targets, such as glycoprotein VI, were reported [1]. In addition, cell therapy is also a promising therapeutic option. Due to its immunomodulatory and regenerative activities, mesenchymal stem cells were also investigated and were used to treat ischemic stroke and retinal injury in preclinical settings [2,3,4]. Thus, it is important to find a new drug or therapeutic approach to prevent thrombosis-relevant events.

The phospholipase D (PLD) enzyme can catalyze the hydrolysis of phosphatidylcholine to choline and phosphatidic acid under a variety of stimuli, such as growth factors and neurotransmitters. Phosphatidic acid is a second messenger that can function in vesicular trafficking, cytoskeleton reorganization, and different signaling pathways [5]. There are two classical PLD isoforms in mammals, PLD1 and PLD2, which were proposed to be involved in many physiological and pathological processes in cancer, immunity, and thrombus formation [5,6,7,8,9]. In mice, the deficiency of PLD1, not PLD2, can impair thrombus formation in pulmonary thromboembolism, aortic occlusion and ferric chloride (FeCl_3_)-induced carotid artery injury models [6]. Thielmann et al. [7] also reported that these two isoforms have partially redundant functions in thrombus formation and that *Pld1^−/−^/Pld2^−/−^* mice display reduced granule release and enhanced integrin activation. Moreover, Stegner et al. reported that pharmacological inhibition of both PLD1 and PLD2 by 5-fluoro-2-indolyl des-chlorohalopemide (FIPI) can diminish granule release as well [8]. Further, these reports showed that platelets from PLD1 or PLD2 knockout mice or FIPI-treated platelets did not show impairment of platelet aggregation. However, our previous study revealed that 5 μM PLD1 inhibitor (VU0155069; VU1) or PLD2 inhibitor (VU0364739; VU2) led to a complete inhibition of platelet aggregation induced by collagen in humans, but not in mice, suggesting that PLD plays differential regulatory roles between mouse and human platelets [9].

Notably, studies revealed different results obtained from *Pld1^−/−^* mice in different animal models. For example, *Pld1^−/−^* mice revealed considerable protection against lethal pulmonary embolization, FeCl_3_-induced injury of carotid artery, and mechanical injury of the abdominal aorta [6], but demonstrated non-significant protection against FeCl_3_-induced injury of small mesenteric arterioles [7]. However, compared to *Pld1^−/−^/Pld2^−/−^* or FIPI (3 mg/kg)-treated mice, *Pld1^−/−^* mice demonstrated similar or even better improvement in infarct size induced by transient middle cerebral artery (MCA) occlusion (MCAO), [6,7,8]. Moreover, our previous study revealed that pharmacological inhibition of PLD1, but not PLD2, affords a protective effect against thrombosis in mesenteric microvessels of mice [9]. These results revealed that PLD1 plays a more crucial role in thrombus formation in mice.

Owing to these diversities, we used the selective pharmacological PLD1 inhibitor VU1 and PLD2 inhibitor VU2 to further determine or confirm the role of PLD1 and PLD2 in thrombosis-relevant events, including pulmonary thrombosis and transient MCAO-induced brain injury.

## 2. Results

### 2.1. Effects of the PLD1 and PLD2 Inhibitors in Adenosine 5′-Diphosphate (ADP)-Induced Acute Pulmonary Thrombosis in Mice

We previously demonstrated that the PLD1 inhibitor VU1 (2.7 mg/kg), but not the PLD2 inhibitor VU2 (2.5 mg/kg), can significantly prolong occlusion time [9]. Here, we further investigated the role of PLD1 and PLD2 in acute pulmonary thrombosis and transient MCAO-induced brain injury in mice.

**Figure 1:**
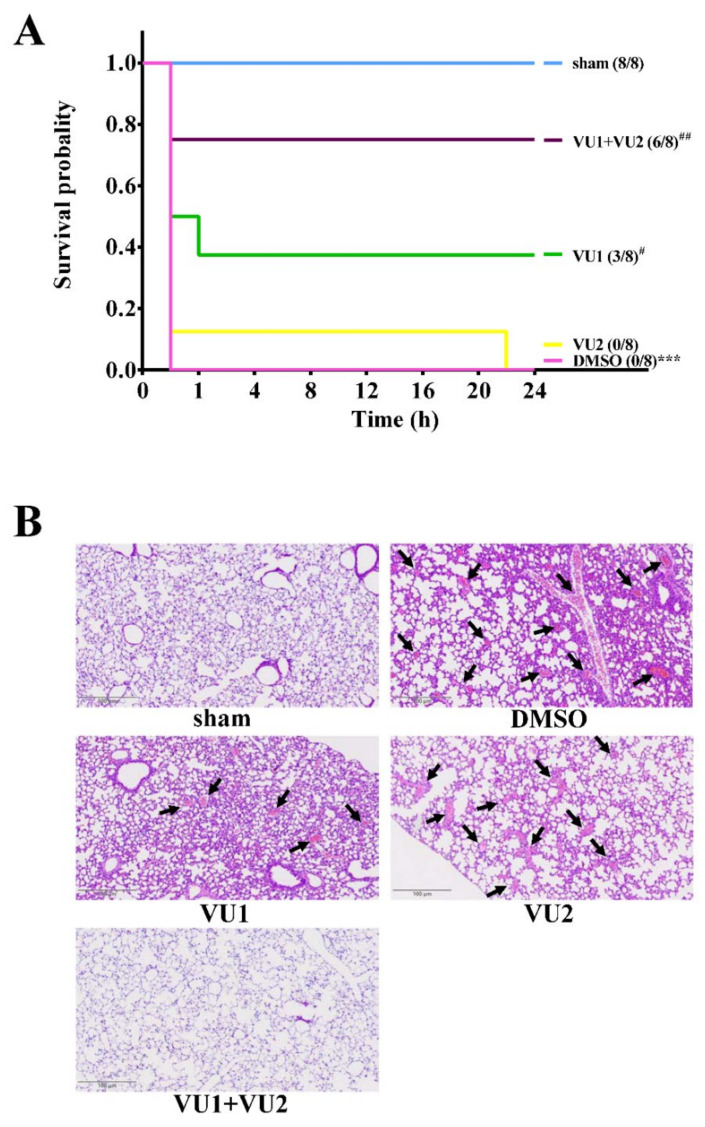
Effects of VU1 and VU2 on adenosine 5′-diphosphate (ADP)-induced acute pulmonary thrombosis. ICR (Institute of Cancer research) mice (male, 5–6 weeks old) were intravenously administered dimethyl sulphoxide (DMSO) (solvent control), PLD1 inhibitor VU1 (2.7 mg/kg), PLD2 inhibitor VU2 (2.5 mg/kg), or co-treatment of VU1 and VU2 for 10 min. ADP (1.4 g/kg) was injected in the tail vein to induce acute pulmonary thrombosis. (**A**) The survival rate was determined within 24 h after ADP injection, and (**B**) pulmonary thrombosis was observed by staining lung tissue sections with hematoxylin-eosin (HE). The arrow indicates occlusive thrombi in pulmonary vessels. The scale bar = 100 μm. The survival rate was plotted using the Kaplan–Meier survival method (*n* = 8); *** *p* < 0.001, compared to the sham-operated group. ^#^ *p* < 0.05 and ^##^ *p* < 0.01, compared to the DMSO group.

As shown in Figure 1A, the data revealed that ADP obviously induced lethal pulmonary thrombosis with a survival rate of 0% (0/8) in the solvent control group, as well as the VU2-treated group, within 24 h after ADP injection, indicating that VU2 alone did not have a protective effect. However, VU1 had a slight but significant protective effect, with a survival rate of 37.5% (3/8, *p* < 0.05). Moreover, co-treatment with VUI and VU2 led to a good survival rate of 75% (6/8; *p* < 0.01), as well as in the aspirin (positive control)-treated group (6/8, *p* < 0.001; Figure S1). These findings revealed that compared to PLD2 inhibition, PLD1 inhibition led to a better improvement in survival rate and that co-treatment with VU1 and VU2 led to a considerable improvement in survival rate. Similar to statistical analysis of survival rate, the histologic graph of lung sections clearly demonstrated that ADP induced considerable pulmonary thrombosis (arrows), as detected by HE staining, in the solvent control groups as well as in the VU2-treated group (Figure 1B). Treatment with VU1 led to a slight improvement, and co-treatment with VU1 and VU2 considerably reduced pulmonary thrombosis. Taken together, these results were in line with those of a previous study that suggested partially redundant roles of PLD1 and PLD2 [7].

### 2.2. Effects of PLD1 and PLD2 Inhibitors on Transient MCAO-Induced Brain Injury

**Figure 2:**
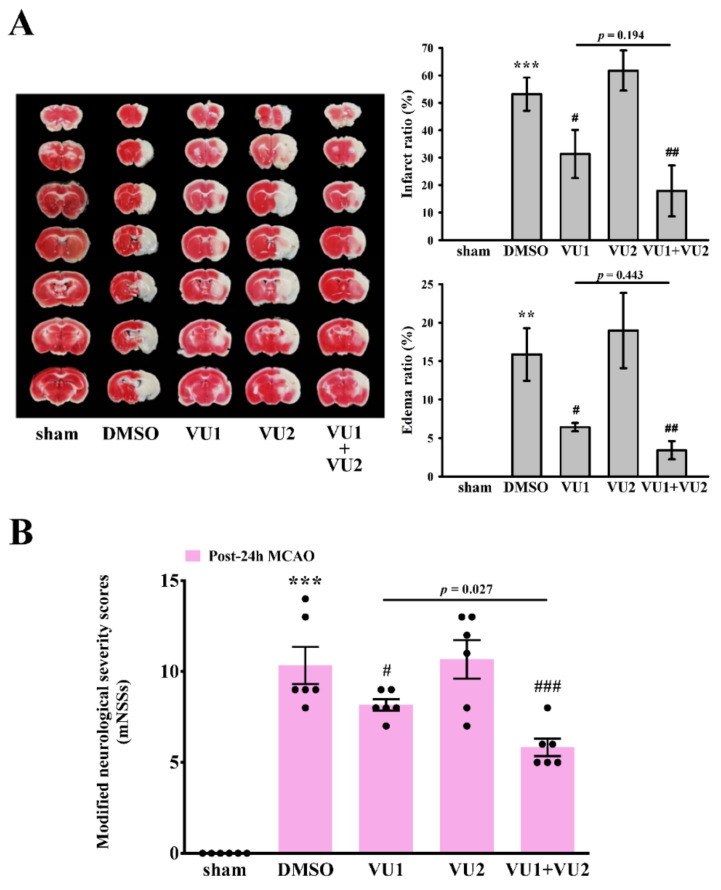
Effects of VU1 and VU2 on transient MCAO-induced brain injury. C57BL/6 mice (male, 5–6 weeks old) were intravenously administrated with DMSO (solvent control), PLD1 inhibitor VU1 (2.7 mg/kg), PLD2 inhibitor VU2 (2.5 mg/kg), or co-treatment of VU1 and VU2 for 30 min. (**A**) Mice were subjected to MCAO for 30 min, followed by 24-h reperfusion. After sacrifice, coronal sections were cut and stained using 2,3,5-triphenyltetrazolium chloride; white areas indicate infarction, and red areas indicate normal tissues (left panel). Edema and infarct ratios (right panel) were calculated through image analysis and are reported as a ratio of the non-ischemic hemisphere. Infarct ratio was corrected for edema. (**B**) Before MCAO or 24 h after MCAO, mNSSs were calculated to evaluate the neurobehavioral function of each mouse. Total mNSS score was 14 (score = 0 means normal; score = 14 means severe deficit). Data are presented as the mean ± standard error of the mean (*n* = 6). ** *p* < 0.01 and *** *p* < 0.001, as compared to the sham-operated group; ^#^ *p* < 0.05, ^##^ *p* < 0.01, and ^###^ *p* < 0.001 as compared to the DMSO group.

We further determined the effect of VU1 and VU2 on transient MCAO-induced brain injury. As shown in Figure 2A, we found that transient MCAO leads to severe brain damage, as detected by infarct size (53.2 ± 6.0%, *p* < 0.001, brain damage; white) and brain edema (15.9 ± 3.4%, *p* < 0.01). However, treatment with VU1 (2.7 mg/kg) significantly reduced infarct size (31.3 ± 8.8%, *p* < 0.05) and brain edema (6.4 ± 0.5%, *p* < 0.05), but VU2 (2.5 mg/kg) treatment did not. Moreover, co-treatment with VU1 and VU2 could reduce the infarct size (17.9 ± 9.2%, *p* < 0.01) and brain edema (3.4 ± 1.2%, *p* < 0.01), as well as in the aspirin-treated group (infarct size, 10.8 ± 7.2%, *p* < 0.001; brain edema, 0.9 ± 0.8, *p* < 0.001; Supplementary Figure S2). Although groups between VU1 alone and a combination of VU1 and VU2 did not show significant difference, combination of VU1 and VU2 showed a better tendency than VU1 in improving infarct size and edema. Taken together, these findings revealed a significant beneficial protection against brain injury, when PLD1 and PLD2 were concurrently inhibited.

### 2.3. Effects of PLD1 and PLD2 Inhibitors on Neurobehavioral Function after Transient MCAO

Subsequently, the modified neurological severity score (mNSS) was performed to evaluate neurological deficit by observing the behavior of mice. Before MCAO, all mice (five groups) had normal neurological function (mNSS score = 0); 24 h after MCAO, the results revealed that the solvent control group showed a severe neurological deficit, with a higher mNSS score (DMSO group: *p* < 0.001; Figure 2B). Moreover, no difference in mNSS scores was observed between the solvent control group and the VU2-treated group, indicating that VU2 alone did not have a protective effect. However, VU1 treatment alone, as well as co-treatment with VU1 and VU2 showed a relatively mild severity, as compared to the solvent control (VU1-treated group: *p* < 0.05; co-treatment group: *p* < 0.001; Figure 2B). Moreover, co-treatment with VU1 and VU2 also showed a significantly better improvement than VU1 alone (*p* = 0.027). Here, we evaluated the severity of neurological deficits in the short-term (24 h after MCAO) but not in a long-term (e.g., 7 or 14 days after MCAO). Therefore, we only observed the severity of neurological deficit, but not with an aim to further evaluate functional recovery on treatment with PLD inhibitors.

**Figure 3:**
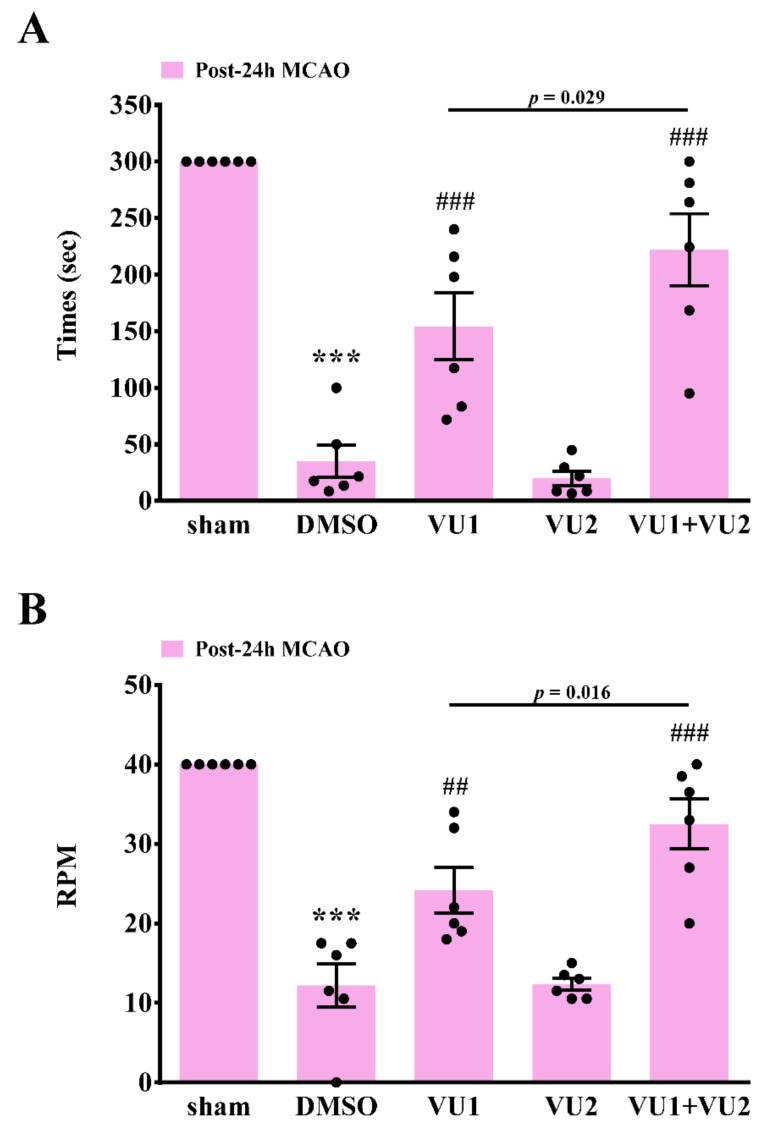
Effects of VU1 and VU2 on post-stroke motor coordination and balance. Before MCAO or 24 h after MCAO, an accelerating rotarod with rotation ranging from 4 to 40 rpm was used to evaluate post-stroke motor coordination and balance of each mouse within 5 min. (**A**) The times (or duration) for which the mice stayed on an accelerating rotarod and (**B**) rpm achieved of mice falling from the rotating drum were recorded. The mice were divided into five groups—sham-operated, DMSO-treated (solvent control), VU1-treated (2.7 mg/kg), VU2-treated (2.5 mg/kg), and VU1+VU2-treated. Data are presented as means ± standard error of the mean (*n* = 6). *** *p* < 0.001, as compared to the sham-operated group; ^##^ *p* < 0.01 and ^###^ *p* < 0.001 as compared to the DMSO group.

**Figure 4:**
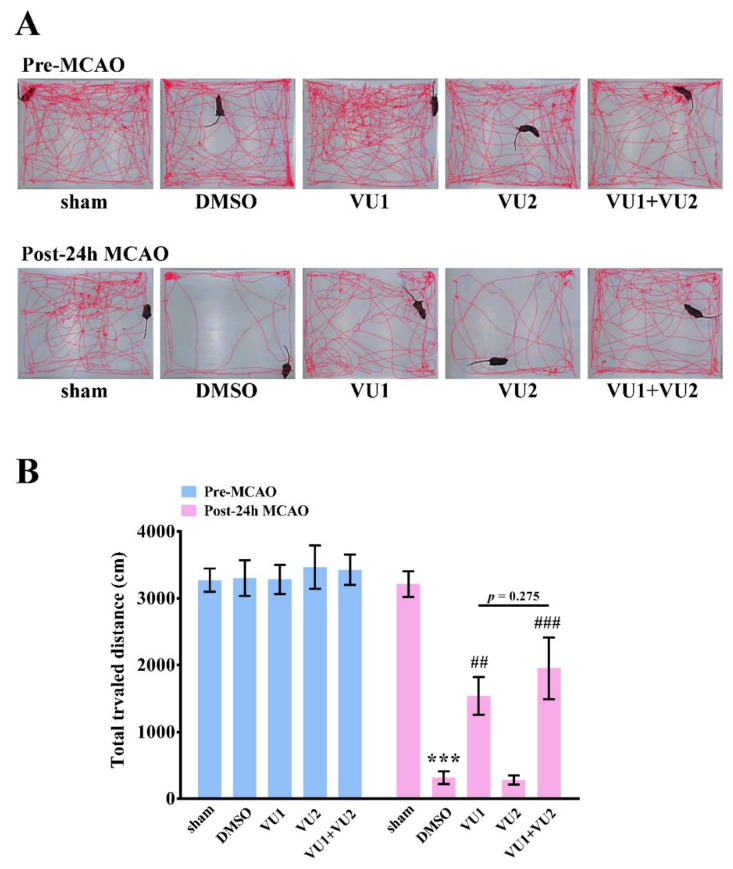
Effects of VU1 and VU2 on post-stroke locomotor activity. Before MCAO or 24 h after MCAO, an open-field test was used to evaluate post-stroke locomotor activity of each mouse for 10 min. The total distance traveled by each mouse in an open arena was (**A**) automatically recorded by a camera and (**B**) analyzed using the video tracking system Ethovision. The mice were divided into five groups—sham-operated, DMSO-treated (solvent control), VU1-treated (2.7 mg/kg), VU2-treated (2.5 mg/kg), and VU1+VU2-treated. Data are presented as means ± standard error of the mean (*n* = 6). *** *p* < 0.001, as compared to the sham-operated group; ^##^ *p* < 0.01 and ^###^ *p* < 0.001 as compared to the DMSO group.

Rotarod and open-field tests were also performed to examine motor function 24 h after MCAO. The rotarod test was widely used to evaluate motor coordination and balance in rodents [10]. As shown in Figure 3, the time duration for which the mice stayed on an accelerating rotarod with rotations ranging from 4 to 40 rpm within 5 min was recorded, and the rpm achieved by mice falling from the rotating drum were also recorded. The data revealed that transient MCAO led to severe motor dysfunction, as determined by a considerable decrease in time duration (Figure 3A) and the rpm (Figure 3B) in the solvent control group (35.2 ± 14.3 s, *p* < 0.001 and 12.2 ± 2.7 rpm, *p* < 0.001, respectively), as compared to the sham control group (300 s and 40 rpm, respectively). The data revealed that treatment with VU1 but not VU2 also showed a marked improvement compared to the solvent control (VU1, 154.5 ± 29.6 s, *p* < 0.001 and 24.2 ± 2.9 rpm, *p* < 0.01, respectively; VU2, 20.0 ± 6.2 s, *p* = 0.608 and 12.3 ± 0.7 rpm, *p* = 0.959, respectively). Co-treatment with VU1 and VU2 considerably and significantly increased the time duration and the rpm achieved, compared to the solvent control (222.2 ± 31.8 s, *p* < 0.001 and 32.5 ± 3.1 rpm, *p* < 0.001, respectively). Furthermore, co-treatment with VU1 and VU2 also significantly showed a better improvement than VU1 alone (*p* < 0.05). Open-field test is widely used to evaluate motor function and normal exploratory locomotion in rodents [10,11]. The data demonstrated no significant differences among the five groups in the total distance traveled before MCAO (Figure 4). After MCAO, the solvent control group (316.9 ± 96.7 cm, *p* < 0.001) showed a considerable reduction in the total distance traveled, indicating that MCAO insults led to severe deficits in locomotor activity, which could be significantly reversed by VU1 (1539.5 ± 282.3 cm, *p* < 0.01) or co-treatment of VU1 and VU2 (1953.5 ± 461.8 cm, *p* < 0.01), but not VU2 alone (281.0 ± 67.7 cm, *p* = 0.137). Although groups between VU1 alone and combination of VU1 and VU2 did not show significant difference, the combination of VU1 and VU2 showed a better tendency than VU1 in locomotor activity. Collectively, these findings revealed that concurrent inhibition of PLD1 and PLD2 afforded optimal protection against MCAO insults.

## 3. Discussion

The significance of the current study was that it further defined the roles of PLD1 and PLD2 in thrombosis-relevant events in mice. Here, we confirmed that PLD1 might play more important roles than PLD2 and that both PLD1 and PLD2 act synergistically, or partially redundantly, in regulating thrombosis-relevant events, including acute pulmonary thrombosis and ischemic stroke, in mice.

PLD is involved in various cell biological processes, such as regulated exocytosis, endocytosis, cell migration, proliferation, apoptosis, and autophagy [12]. PLD also participates in pathological processes, such as cancer and Alzheimer’s disease [13,14,15,16]. However, the role of PLD in thrombosis remains unclear. Deficiency of PLD1, but not PLD2, was reported to impair thrombus formation in the models of pulmonary thromboembolism, aortic occlusion, and FeCl_3_-induced carotid artery injury [6]. Moreover, PLD1 is a regulator of platelet-mediated inflammation [17]. This report also showed that adhesion of PLD1-deficient but not PLD2-deficient platelets on activated endothelial cells obviously decreased under high shear rates, suggesting that PLD1 plays a major role in platelet-mediated inflammation under high shear rates [17]. In addition, another study reported that PLD1 could regulate LPS-induced sepsis, which might be due to the reduced thrombin generation on PLD1-deficient platelets and the subsequent reduced fibrin formation and platelet consumption, eventually reducing the risk of disseminated intravascular coagulation [18]. By contrast, Thielmann et al. reported that *Pld1^−/−^* mice did not show a significant protection against thrombus formation in small mesenteric arterioles induced by FeCl_3_, as compared to wild-type mice, but *Pld1^−/−^/Pld2^−/−^* mice demonstrated a prolonged time of full occlusion. Moreover, *Pld1^−/−^/Pld2^−/−^* mice displayed reduced granule release and enhanced integrin activation. Thus, the authors suggest that these two isoforms show partially redundant functions in thrombus formation and granule release [7]. These two studies using PLD knockout mice revealed a discrepancy in the role of PLD1 among several different thrombotic models of mice. This needs to be clarified in further research.

Our previous study using the selective pharmacological inhibitor of PLD1 VU1 (2.7 mg/kg) or of PLD2 VU2 (2.5 mg/kg) also demonstrated that only PLD1 inhibition, but not PLD2 inhibition, could significantly delay thrombus formation, indicating that PLD1 might be more crucial than PLD2 in the thrombotic events [9]. Therefore, in the present study, we further used two different thrombotic models of acute pulmonary thrombosis and ischemic stroke, to define the roles of PLD1 and PLD2. The results revealed that only PLD1 inhibition, but not PLD2 inhibition, could partially improve pulmonary thrombosis-induced death, which is consistent with the results reported by Elvers et al. [6]. Moreover, simultaneous PLD1 and PLD2 inhibition led to a considerable improvement in the survival rate of mice. Likewise, only PLD1 inhibition, but not PLD2 inhibition, could partially improve ischemic stroke, and inhibition of both PLD1 and PLD2 could afford considerable protection against ischemic stroke. Post-stroke behavior of the mice was also evaluated using mNSS, rotarod test, and the open-field test. These tests also revealed that the inhibition of either PLD1 or both PLD1 and PLD2 affords protective effects against neurological deficit after stroke. This finding was also in agreement with that of a previous study, which showed that the pharmacological inhibition of both PLD1 and PLD2 by FIPI could effectively prevent ischemic stroke [8]. Although we did not exclude the possibility that pharmacological inhibitors might have off-target effects, our previous [9] and present study revealed rather consistent findings that inhibition of only PLD1, but not PLD2, could afford a protective effect against thrombosis-relevant events and that enhanced protective effects appeared when PLD1 and PLD2 inhibitors were used simultaneously. These results were also consistent with those of previous studies in which PLD-knockout mice and nonselective PLD inhibitor FIPI were used [6,7,8]. However, among all of these studies (including ours) that investigated the role of PLD, only one study showed that the absence of PLD1 was not resistant to thrombus formation in small mesenteric arterioles induced by 20% FeCl_3_ [7]. In general, PLD1, therefore, was involved in thrombus formation.

We previously demonstrated that PLD1 and PLD2 are essential for maintaining platelet activation in humans [9]. However, other studies reported that PLD1 is required for full integrin activation of platelets [6] and that it plays a major role in regulating platelet-mediated inflammation [17], but absence of PLD2 has no effect on platelet activation [7] in mice. Thus, PLD has different regulatory effects on platelet activation in humans and mice. This discrepancy might be attributed to the difference between the two species. Meanwhile, genetic deletion or pharmacological inhibition of PLD1, PLD2, or both of PLD1 and PLD2, did not result in a prolonged bleeding time, suggesting that PLD inhibition might be a safe strategy to prevent cardiovascular diseases [9,17,18,19]. However, in the current study, there is a limitation that we cannot exclude the possibility of off-target effect of inhibitors. It is hard to find the volunteers with PLD1 or PLD2 deficiency to validate the difference between mice and human. Moreover, previous studies suggested that it is crucial to understand the differences between humans and mice [20,21]. This might also contribute to causality of the failure of clinical trials, which is inconsistent with the results of the preclinical study. Therefore, the PLD enzyme might need extensive investigation to understand the difference of PLD enzyme between mice and humans in the future. Moreover, targeting PLD might provide a therapeutic strategy to develop a novel antiplatelet drug. Nowadays, every scientist is dedicated to finding a drug or an approach to prevent a stroke or heart attack, but also hopes that this drug does not affect hemostasis [1]. This issue is critical because the side effect of bleeding remains a limitation of clinical antiplatelet drugs, which has limited their use.

Excepting platelet-mediated inflammation and the thrombotic events in mouse models in which PLD1 was reported to play a major role, we previously demonstrated that both PLD1 and PLD2 are crucial regulators in human platelet activation [9]. Similarly, both PLD1 and PLD2 reportedly regulate macrophage phagocytosis [22], Alzheimer’s disease [16], and tumor growth [23,24]. Collectively, PLD isoforms might play different roles in different species and diseases that involve corresponding cells or tissues.

On the other hand, cell therapy is also a promising therapeutic option in ischemic stroke. For example, dental pulp stem cells (DPSCs) were reported to provide a tempting prospect for stroke treatment [25]. However, the safety issues of DPSCs, such as an in vitro expansion and in vivo delivery are a concern. It is important to use quality-controlled, and potentially advantageous supplements to establish a preparatory study for regenerative medicine applications [26], such as stroke treatment.

## 4. Materials and Methods

### 4.1. Materials

VU0155069 (VU1) and VU0364739 (VU2) were purchased from Tocris Bioscience (Bristol, UK). Adenosine 5′-diphosphate (ADP) and 2,3,5-triphenyltetrazolium chloride (TTC) were purchased from Sigma (St. Louis, MO, USA). We purchased 6–0 monofilament nylon sutures coated with silicon from Doccol Corp. (Sharon, MA, USA). VU1 and VU2 were dissolved in dimethyl sulphoxide (DMSO) and stored at 4 °C until use.

### 4.2. Animals

Mice (ICR and C57BL/6; 20–25 g, male, 5–6 weeks old) were purchased from BioLasco (Taipei, Taiwan). All procedures were conducted in accordance with the Guide for the Care and Use of Laboratory Animals (Eighth Edition, 2011) and approved by the Affidavit of Approval of Animal Use Protocol of Taipei Medical University (Approval No. LAC-2016-0195, 21 Jul 2016). Mice doses VU1 (2.7 mg/kg) and VU2 (2.5 mg/kg) was calculated according to the body surface area normalization method [9,27]. VU1 and VU2 were intravenously administered from the tail vein, in all experiments.

### 4.3. Acute Pulmonary Thrombosis Induced by ADP in Mice

This experiment was performed according to the method that Lien et al. reported [28]. In brief, mice were intravenously injected with ADP (1.4 g/kg) to induce acute pulmonary thrombosis. Twenty-four hours after ADP injection, the survival rate of each group was determined. Then, all survivors were sacrificed by CO_2_ in a chamber. The lung was removed from the body, fixed with 4% formalin, and embedded in paraffin. Afterwards, paraffin sections of the lung were stained with hematoxylin-eosin (HE). The stained lung sections were further observed and digitalized using the Microvisioneer Manual Whole Slide Imaging (microvisioneer.com; Josef Bauer, Freising, Germany). The mice were divided into five groups—sham-operated, DMSO-treated (solvent control), VU1-treated (2.7 mg/kg), VU2-treated (2.5 mg/kg), and VU1+VU2-treated. All treatments were administered 10 min before ADP administration for all groups, except for the sham-operated group.

### 4.4. Transient MCAO-Induced Brain Injury in Mice

This method to induce ischemic stroke was described previously [28]. In brief, mice were anesthetized with a mixture of 75% air and 25% oxygen containing 3% isoflurane. To occlude the right MCA, a 6–0 monofilament nylon suture coated with silicon was introduced and gently advanced from the external to internal carotid artery lumen, until it could no longer be advanced. Thirty minutes after MCAO, the suture was gently withdrawn to restore blood supply. Twenty-four hours after reperfusion, all groups of mice were euthanized through decapitation. The brains were removed and cut into 1-mm coronal slices. The brain infarct size and edema were observed after brain sections were stained with 2% TTC. The TTC-unstained infarct areas were calculated by an image analyzer (Image-Pro Plus, Rockville, MD, USA) and then summed to obtain the total infarct volume (in mm^3^) of a brain. Infarct volumes were expressed as a percentage of the contralateral hemisphere volume using the formula (the area of the intact contralateral [left] hemisphere—the area of the intact region of the ipsilateral [right] hemisphere) to compensate for edema formation in the ipsilateral hemisphere. The mice were divided into five groups—sham-operated, DMSO-treated (solvent control), VU1-treated (2.7 mg/kg), VU2-treated (2.5 mg/kg), and VU1+VU2-treated. All treatments were administered 30 min before MCAO, except for the sham-operated group.

### 4.5. Modified Neurological Severity Scores

Modified neurological severity scores (mNSSs), including a composite of motor, reflex, and balance tests, was graded on a scale of 0–14 (normal score 0; maximal deficit score 14) [29], where a higher score indicated a more severe injury. mNSSs were calculated for each mouse immediately before MCAO and at 24 h after MCAO.

### 4.6. Rotarod Test

The rotarod test was performed to evaluate motor coordination and balance [10]. Mice in all five groups were trained to stay on the rotarod apparatus for 3 consecutive days prior to any test. They were trained on an accelerating rotarod (4–40 revolutions per min [rpm] over 5 min with a gradually increasing speed of 4 rpm every 30 s) (Ugo Basile, Varese, Italy). Before MCAO and 24 h after MCAO, mice were subjected to the rotarod test, and the time duration for which each mouse stayed on the rotarod and the speed achieved by each mouse falling from the rotating drum were recorded [30].

### 4.7. Open-Field Test

The open-field test is widely used to evaluate motor function and normal exploratory locomotion in rodents [10,11]. The locomotor activity of the mice in all five groups was measured through the open-field test. In brief, the mice were placed at the center of a rectangular testing chamber (40 × 40 × 40 cm^3^), with the video camera mounted on the top of the box for recording the exploratory behavior of the mice. The mice were allowed to freely explore the arena for 10 min. The total distance traveled by the mice was analyzed using the video tracking system EthoVision (Noldus Information Technology, Wageningen, Netherlands). The box was cleaned with 75% ethanol between trials to avoid interference of the analyses between different testing animals [31].

### 4.8. Data Analysis

All values are expressed as means ± SEM. Statistical analysis was performed by analysis of variance (ANOVA) followed by the Newman–Keuls method. For survival analysis, survival curves were plotted using the Kaplan–Meier curves and analyzed using the log-rank test, and all pair-wise multiple comparison procedures were performed by the Holm-Sidak method. A *p* of < 0.05 was considered to be statistically significant.

## 5. Conclusions

Taken together, this study further confirmed that PLD1 might play a more crucial role than PLD2 in thrombus formation. Moreover, PLD1 and PLD2 can act synergistically or have partially redundant functions in regulating thrombus formation.

## Supplementary Materials

Supplementary materials can be found at https://www.mdpi.com/1422-0067/21/18/6954/s1, Figure S1 and Figure S2.

## Abbreviations

PLD	Phospholipase D
FeCl_3_	Ferric chloride
FIPI	5-fluoro-2-indolyl des-chlorohalopemide
MCA	Middle cerebral artery
MCAO	Middle cerebral artery occlusion
ADP	Adenosine 5′-diphosphate
TTC	2,3,5-triphenyltetrazolium chloride
DMSO	Dimethyl sulphoxide
HE	Hematoxylin-eosin
mNSSs	Modified neurological severity scores
rpm	Revolutions per min
