# Supplementary material for: Phospholipase D1 and D2 Synergistically Regulate Thrombus Formation
Source: Int J Mol Sci. 2020 Sep 22;21(18):6954. doi: 10.3390/ijms21186954 (PMC7555624; doi:10.3390/ijms21186954)
Supplement: Supplementary file 1 [file ijms-21-06954-s001.pdf]

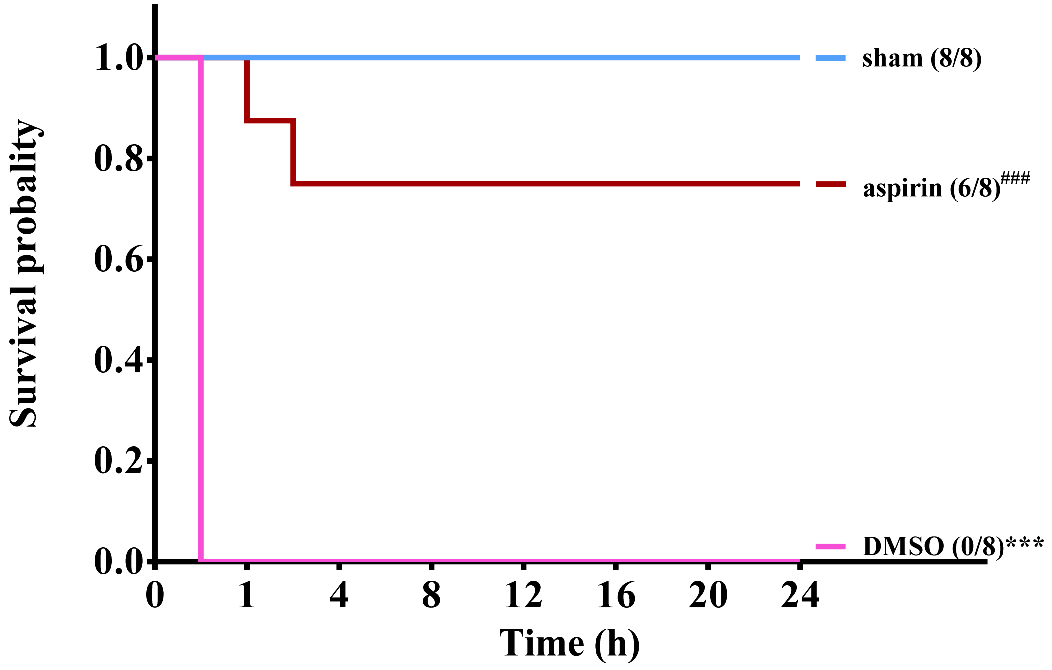

Supplemental Figure 1. Effects of aspirin on ADP-induced acute pulmonary thrombosis. ICR mice (male, 5–6 weeks old) were intravenously administered DMSO (solvent control), or aspirin (20 mg/kg) for 10 min. ADP (1.4 g/kg) was injected in the tail vein to induce acute pulmonary thrombosis. The survival rate was determined within 24 h after ADP injection. The survival rate was plotted using the Kaplan–Meier survival method (n = 8). ***$p < 0.001$, compared with the sham-operated group. ###$p < 0.001$, compared with the DMSO group.

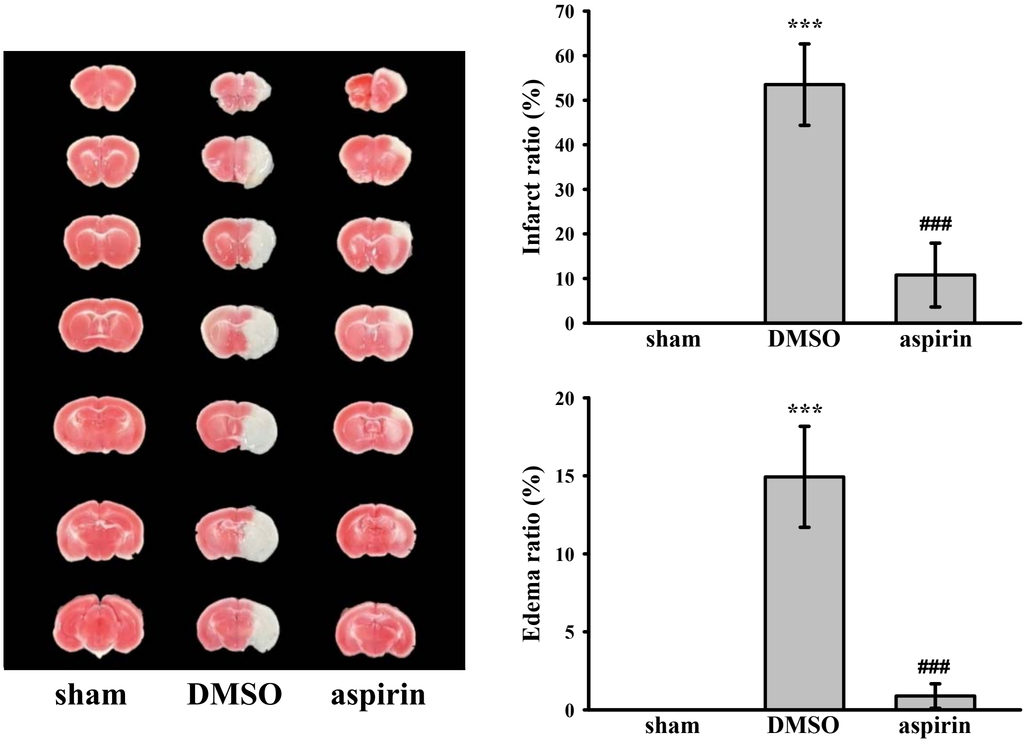

Supplemental Figure 2. Effects of aspirin on transient MCAO-induced brain injury. C57BL/6 mice (male, 5–6 weeks old) were intravenously administrated with DMSO (solvent control), or aspirin (20 mg/kg) for 30 min. Mice were subjected to MCAO for 30 min followed by 24-h reperfusion. After sacrifice, coronal sections were cut and stained with 2,3,5-triphenyltetrazolium chloride; white areas indicate infarction, and red areas indicate normal tissues (left panel). Edema and infarct ratios (right panel) were calculated through image analysis and are reported as a ratio of the non-ischemic hemisphere. Infarct ratio was corrected for edema. Data are presented as the mean ± standard error of the mean (n = 6). ***$p < 0.001$, compared with the sham-operated group; ###$p < 0.001$ compared with the DMSO group.
